# Hypertension-mediated organ damage and established cardiovascular disease in patients with hypertension: the China Hypertension Survey, 2012–2015

**DOI:** 10.1038/s41371-021-00635-z

**Published:** 2021-11-19

**Authors:** Xin Wang, Guang Hao, Lu Chen, Ying Yang, Haoqi Zhou, Yuting Kang, Lance Shaver, Zuo Chen, Congyi Zheng, Linfeng Zhang, Suning Li, Zengwu Wang, Runlin Gao

**Affiliations:** 1 Division of Prevention and Community Health, National Center for Cardiovascular Disease, National Clinical Research center of Cardiovascular Disease, State Key Laboratory of Cardiovascular Disease, Fuwai Hospital, Peking Union Medical College & Chinese Academy of Medical Sciences, Beijing, China; 2 Department of Epidemiology, School of Medicine, Jinan University, Guangzhou, China; 3 Faculty of Medicine, University of British Columbia, Vancouver, BC Canada; 4 Department of Cardiology, Fuwai Hospital, Peking Union Medical College & Chinese Academy of Medical Sciences, Beijing, China

**Keywords:** Cardiovascular diseases, Disease prevention

## Abstract

Hypertension is a major health burden worldwide. However, there is limited data on the status of hypertension-mediated organ damage (HMOD) and established cardiovascular (CV) disease in Chinese hypertensive patients. The aim of this study is to determine the prevalence of HMOD and established CV disease in a nationally representative population in China. A stratified multistage random sampling method was used in the China Hypertension Survey and 21,243 participants aged 35 or older were eligible for analysis in this study. For each participant, the demographic information and a self-reported medical history were acquired. Blood pressure was measured with the electronic device 3 times on the right arm, supported at heart level, after the participant was sitting at rest for 5 min. Samples of blood and urine were tested. 2-D and Doppler echocardiography were used to assess the heart’s function and structures. Sampling weights were calculated based on the 2010 China population census data. Overall, the weighted prevalence of asymptomatic HMOD was 22.1%, 28.9%, 23.1%, 6.4%, and 6.2% for wide pulse pressure, left ventricular hypertrophy, microalbuminuria, chronic kidney disease, and abnormal ankle-brachial index, respectively. For the established CV disease, the weighted prevalence was 1.8%, 1.3%, 2.0%, and 1.1% for stroke, coronary artery disease, heart failure, and atrial fibrillation, respectively. The prevalence of asymptomatic HMOD and established CV disease was greater with higher blood pressure level (*P* < 0.05), rather than ankle-brachial index. Compared to those with uncontrolled hypertension, the prevalence of asymptomatic HMOD was lower in patients with controlled hypertension. In summary, the prevalence of HMOD in Chinese people aged 35 or older was very common, indicating a substantial future burden of both morbidity and mortality from hypertension in China. Clinical trial registration number: ChiCTR-ECS-14004641.

## Introduction

The global prevalence of hypertension was estimated to be 1.13 billion in 2015, with an alarming number of 235 million in east Asia [1]. Hypertension prevalence has been rising in China in recent decades, with up to 23.2% prevalence in Chinese people aged 18 and older, or an estimated 244.5 million individuals [2], resulting in an increase of blood pressure (BP)-related morbidity and mortality [3]. If the definition of hypertension from the 2017 ACC/AHA guideline was adopted, the prevalence of hypertension would be double, suggesting a critical burden of elevated BP in China. Furthermore, the prevalence of hypertension is expected to continue increasing with rapid economic development, urbanization, and aging.

Hypertension is the leading cause of cardiovascular (CV) disease globally [4]. It is well known that the incidence of several CV diseases, including stroke, coronary heart disease, and peripheral artery disease, is closely linking to hypertension. Meanwhile, many patients with long-term elevated BP may present with hypertension-mediated organ damage (HMOD, previously termed target organ damage), and at first without symptoms [5], including left ventricular hypertrophy (LVH), microalbuminuria, and so on, many of which are usually markers of preclinical cardiovascular disease (CVD).

Identifying HMOD could help to estimate the burden of high-CV-risk populations in the hypertensive population. Furthermore, earlier screening and intervention in HMOD may lead to more effective therapeutic strategies and reduce future CV events [5–7]. Since the various classes of antihypertensive agents have protective actions on end-target organs [5, 8, 9], the distribution of HMOD and CVD may differ in hypertensive patients depending on whether they use antihypertensive drugs or not. However, there is limited data on the status of HMOD and established CVD in China and worldwide. Therefore, the aim of this study is to determine the prevalence of HMOD and established CVD in a nationally representative population in China.

## Methods

### Survey participants

The China Hypertension Survey was conducted between October 2012 and December 2015, and the design was published previously [10, 11]. Briefly, a stratified, multistage, random sampling method was used to obtain a nationally representative sample of the general Chinese population aged 15 years or older. All 31 provinces in mainland China were covered in this survey. For this sub-study, as previously published [12], all selected urban and rural areas were stratified into eastern, middle and western regions again according to both geographical locations as well as economic strata. Using a simple random sampling method, 16 cities and 17 counties were selected. Then, at least three communities or villages were randomly selected from each city/county. To meet the designed sample size of 35,000 participants aged ≥35 years and to take nonresponse into account in the survey, 56,000 subjects were randomly selected and invited. Among these, 34,994 responded positively with a response rate of 62.5%. All the participants were examined with blood samples, electrocardiograms, and echocardiograms data. However, 6150 participants were excluded due to missing echocardiographic data, 3757 participants due to missing data on biomarkers, and 3844 individuals due to missing data on educational attainment, coronary artery disease (CAD), diabetes, peripheral artery disease (PAD), heart failure (HF), and stroke. A total of 21,243 individuals were eligible for analysis. Supplementary Table 1 showed the differences between the participants who were included and those who were not included in the subsequent analyses.

Written informed consent was obtained from each participant before data collection. The ethics committee of Fuwai Hospital approved the study.

### Measurements

A standardized questionnaire developed by the coordinating center, Fuwai Hospital (Beijing, China), was administered by trained staff to obtain information on demographic characteristics and socioeconomic factors. Height was measured without shoes using a standard right-angle device and a fixed measurement tape (to the nearest 0.5 cm), and weight was measured without heavy clothing using an OMRON body fat and weight measurement device (V-body HBF-371, OMRON, Kyoto, Japan). BMI was calculated as the weight divided by height squared (kg/m^2^) for each participant. BP was measured 3 times on the right arm supported at heart level with the OMRON HBP-1300 professional portable BP monitor (OMRON, Kyoto, Japan) after the participant was sitting at rest for 5 min, with 30 s between each measurement. The accuracy of the Omron HBP-1300 for BP measurement had been verified in our prior study [13]. The echocardiograms were obtained using a commercially available Doppler ultrasonography, with a 3.0 MHz transducer using M-mode, 2-dimensional, spectral Doppler, and color Doppler transthoracic echocardiography with participants in the supine position. It was performed by a certified sonographer, who received a 1-month training before the study on the standardization of quantifying cardiac chambers. For each participant, a self-reported history of CVD was acquired. Peripheral blood samples were collected after an 8–10 h overnight fast and stored at −80 °C, while urine samples in the morning were collected and stored at −25 °C. Albuminuria, blood glucose, and cholesterol were measured by professional medical testing institution.

### Survey outcome definitions

Overweight was defined as a body mass index (BMI) between 24.0 kg/m^2^ and 27.9 kg/m^2^, and obese was defined as a BMI of 28.0 kg/m^2^ or more [14]. BP was categorized as normal (systolic BP (SBP) < 120 and diastolic BP(DBP) < 80), normal high (SBP 120–139 and/or DBP 80–89) in a normal population, Grade 1 (SBP 140–159 and/or DBP 90–99), Grade 2 (SBP 160–179 and/or DBP 100–109), and Grade 3 (SBP ≥ 180 and/or DBP ≥ 110) in patients with hypertension according to the 2010 Chinese guidelines [15]. Awareness of hypertension was defined as self-report of any previous diagnosis of hypertension by a doctor. Controlled hypertension was defined as SBP < 140 mmHg and DBP < 90 mmHg in patients. Participants with a fasting plasma glucose level ≥7.0 mmol/l and/or who were receiving antidiabetic medications were defined as having diabetes. Dyslipidemia was defined as meeting any one of the following four conditions: total cholesterol ≥6.2 mmol/L; triglycerides ≥2.3 mmol/L; low levels of high-density lipoprotein cholesterol <1.0 mmol/L); or high levels of low-density lipoprotein cholesterol ≥4.1 mmol/L [16].

Asymptomatic HMOD was defined by wide pulse pressure ≥60 mmHg in older adults (aged ≥ 65 years), LVH (left ventricular mass index >50 g/m^2^ in men and >47 g/m^2^ in women), microalbuminuria (urine albumin/creatine ratio between 30 and 300 mg/g), chronic kidney disease (CKD), or an abnormal ankle-brachial index (ABI) < 0.9. CKD was defined as either decreased estimated glomerular filtration rate (eGFR; eGFR < 60 ml/min/1.73 m^2^) or albuminuria (urinary albumin to creatinine ratio ≥300 mg/g) according to KDIGO guidelines [17]. Moderate CKD was defined as eGFR between 30 and 59 mL/min/1.73 m^2^ and severe CKD was defined as as GFR < 30 mL/min/1.73 m^2^.

CV events, including stroke, CAD, HF and atrial fibrillation (AF), were defined based on self-reported history, which was verified with medical or hospital records. The stroke included subarachnoid hemorrhage, or a hemorrhagic or ischemic lesion in the brain. CAD was defined as previous having a myocardial infarction, or surgery for coronary revascularization. Heart failure was diagnosed according to the recommendations of the European Society of Cardiology [18, 19], with minor modifications, and defined as follows: patients with a self-reported history of HF; ejection fraction (EF) <50% and at least 6 HF symptoms; or EF ≥ 50% with moderate/severe diastolic dysfunction and at least six HF symptoms [11]. AF is diagnosed by the electrocardiograph (ECG) report or a verified AF history (possible paroxysmal AF) [20].

### Statistical analysis

Variables were summarized using means for continuous data, and with frequencies, percentages, and proportions for categorical data. Two-tailed Student’s *t* tests and ANOVA were used to compare continuous variables and chi-square tests were used to compare categorical variables. All 95% confidence intervals (CI) for the parameters were estimated. A two-sided *P* < 0.05 was considered significant. Two multiple logistic regression analyses were performed to identify factors associated with HMOD and CVD. In each analysis, the following independent variables were included: age group (with 10-year intervals), sex, education, urban/rural, BMI, diabetes, dyslipidemia, and the control status of hypertension. Only the characters with *β* ≥ 0.5 were shown in the forest plot. Statistical analyses were conducted with SAS version 9.4 (SAS Institute INC, Cary, NC, USA) and Stata 14 (STATA Corp., TX, USA).

To represent the national population, sampling weights were calculated based on the 2010 China population census data, and the sampling scheme included an oversampling for specific age or geographic subgroups, nonresponse, and other demographic or geographic differences between the sample and the total population [21]. Adjustment for differential probabilities of selection and the complex sampling design was used to enhance the representativeness of the survey sample population. A comparison was made between participants included and excluded (Supplementary Table 1), and a sensitivity analysis under the missing at random assumption was carried out using multiple imputation.

## Results

A total of 21,243 participants aged 35 years or older (50.2% women and 64.8% from rural areas) were included in the analysis. The hypertensive patients on antihypertensive medications tended to be older, women, overweight/obesity, with higher education levels, and with more complications (Table 1).

**Table 1:** Characteristics of study participants.

Characteristics	Normal population	Patients not aware of hypertension	Patients aware of hypertension		*P* value
			Not on medication	On medication	
*n* (%)	12,368 (58.2)	3872 (18.2)	715 (3.4)	4288 (20.2)	
Age—year	48.7 (48.0–49.4)	55.6 (54.3–56.8)	54.7 (53.1–56.3)	59.5 (58.4–60.6)	<0.001
Female	51.1 (47.8–54.3)	44.6 (38.6–50.8)	36.5 (30.1–43.4)	53.1 (49.6–56.5)	<0.001
Body mass index—kg/m^2^	24.0 (23.7–24.2)	25.0 (24.5–25.5)	25.5 (24.9–26.2)	25.9 (25.4–26.5)	<0.001
<18.5	3.0 (2.2–4.0)	2.3 (1.5–3.5)	1.2 (0.7–2.0)	1.3 (0.7–2.4)	<0.001
18.5–23.9	49.5 (46.2–52.9)	37.5 (33.5–41.7)	32.3 (27.3–37.7)	28.2 (23.8–33.1)	
24.0–27.9	36.7 (34.3–39.2)	41.2 (38.9–43.6)	45.3 (41.2–49.4)	43.4 (41.0–45.7)	
≥28.0	10.7 (9.0–39.2)	18.9 (15.2–23.4)	21.2 (15.2–28.8)	27.2 (22.6–32.3)	
Education attainment					
Elementary school	44.0 (35.4–53)	50.2 (42.2–58.2)	48.8 (36.6–61.2)	50.3 (37.4–63.3)	0.038
Middle high school	49.9 (42.5–57.3)	46.2 (39–53.6)	49.3 (37.5–61.2)	44.8 (34.0–56.1)	
High school or above	6.1 (3.6–10.2)	3.6 (2.3–5.5)	1.8 (0.9–3.9)	4.8 (2.8–8.3)	
Rural	67.3 (48.7–81.7)	63.5 (39.6–82.2)	66.7 (47–81.9)	55 (33.1–75.1)	0.055
Diabetes	5.6 (5.0–6.2)	9.7 (8.4–11.1)	12.2 (8.9–16.4)	17.8 (16.2–19.5)	<0.001
Dyslipidemia	31.5 (30.3–32.8)	37.6 (35.2–39.9)	41.0 (35.5–46.5)	44.3 (42.0–46.6)	<0.001

All values were weighted to represent the total Chinese population aged 35 years or older based on 2010 Chinese census data. Data are represented as percentage (95% CI).

Among Chinese adults with hypertension, the weighted prevalence of asymptomatic HMOD was 22.1%, 28.9%, 23.1%, 6.4%, and 6.2% for wide pulse pressure, LVH, microalbuminuria, CKD, and abnormal ABI, respectively. For established CVD, the weighted prevalence was 1.8%, 1.3%, 2.0%, and 1.1% for stroke, CAD, HF, and AF, respectively. The prevalence of HMOD, especially for wide pulse pressure, LVH, and CKD were significantly higher in women, however, the prevalence of established CV disease was higher in men. There was no significant difference between residents living in an urban or rural area for asymptomatic HMOD and established CVD (Table 2). As expected, the prevalence of asymptomatic HMOD, as well as established CVD, was greater with older age (*P* < 0.05, Supplementary Tables 2, 3).

**Table 2:** The weighted prevalence of hypertension-mediated organ damage and cardiovascular diseases in hypertensive patients by sex and region.

Diseases	Region			Sex			Total
	Urban	Rural	*P* value for region	Men	Women	*P* value for sex	
Asymptomatic HMOD							
Pulse pressure (in older people) ≥60 mmHg	20.0 (16.4–24.2)	23.6 (19.6–228.0)	0.185	18.3 (15.7–221.1)	26.4 (22.2–231.0)	<0.001	22.1 (19.4–225.1)
Left ventricular hypertrophy	24.8 (20.3–230.0)	31.6 (20.7–244.9)	0.163	21.9 (15.3–230.4)	36.6 (29.9–243.8)	<0.001	28.9 (22.7–236.0)
Microalbuminuria	24 (17.9–231.4)	22.5 (20.3–224.8)	0.292	21.1 (17.8–224.8)	25.3 (22.3–228.5)	0.241	23.1 (20.3–226.1)
Moderate chronic kidney disease	6.4 (4.2–29.7)	5.9 (2.5–213.3)	0.650	5.1 (3.0–28.7)	7.3 (4.5–211.5)	0.006	6.1 (3.7–29.9)
Severe chronic kidney disease	0.1 (0.0–20.3)	0.4 (0.2–20.7)	0.112	0.1 (0.0–20.2)	0.5 (0.3–20.8)	0.003	0.3 (0.2–20.5)
Ankle-brachial index <0.9	4.9 (3.9–26.3)	6.9 (3.3–214.2)	0.855	5.0 (2.7–28.9)	7.5 (4.7–211.5)	0.001	6.2 (3.8–29.8)
At least one asymptomatic HMOD	54.9 (48.1–261.5)	60.0 (50.6–268.8)	0.338	50.6 (44.1–257.0)	66.2 (60.5–271.4)	<0.001	58.0 (52.4–263.4)
Established CV disease							
Stroke	1.9 (0.3–211.2)	1.8 (1.0–23.2)	0.740	2.0 (0.9–24.5)	1.7 (0.8–23.5)	0.095	1.8 (0.8–24.0)
Coronary artery disease	2.1 (1.1–23.8)	0.8 (0.4–21.6)	0.057	1.6 (1.0–22.6)	1.0 (0.5–21.9)	0.007	1.3 (0.8–22.1)
Heart failure	2.5 (1.4–24.4)	1.6 (0.6–24.3)	0.373	2.0 (1.0–23.9)	1.9 (1.0–23.6)	0.648	2.0 (1.1–23.5)
Atrial fibrillation	0.8 (0.5–21.3)	1.3 (0.7–22.2)	0.620	1.1 (0.8–21.6)	1.0 (0.6–21.6)	0.042	1.1 (0.8–21.5)
At least one established CV disease	6.7 (5.6–27.9)	5.2 (4.3–26.1)	0.047	6.2 (5.1–27.4)	5.3 (4.5–26.3)	0.254	5.8 (5.1–26.5)

Data are represented as percentage (95% CI), unless otherwise indicated. All values were weighted to represent the total population of Chinese aged 18 years or older based on Chinese census 2010.

*CV* cardiovascular, *HMOD* hypertension-mediated organ damage.

Overall, the prevalence of asymptomatic HMOD, as well as established CVD, was significantly higher in participants with hypertension. The patients who were aware of having hypertension had a higher prevalence of established CVD compared to those who were not aware of having hypertension (8.7% vs. 2.7%, *P* < 0.001). Furthermore, among the patients who were aware of having hypertension, the prevalence of established CVD was higher in those with antihypertensive medicines than those without antihypertensive medicines (9.5% vs. 4.7%, *P* < 0.001). The details of the prevalence of asymptomatic HMOD and established CV disease were presented in Table 3. Sensitivity analysis based on multiple imputation remained the similar (Supplementary Table 4).

**Table 3:** The weighted prevalence of hypertension-mediated organ damage and cardiovascular diseases by with or without hypertension.

Diseases	Normal population	Patients not aware of hypertension	Patients aware of hypertension		Hypertension	*P* value^#^
			Not on medication	On medication		
Asymptomatic HMOD						
Pulse pressure (in older people) ≥60 mmHg	2.4 (2.0–3.0)*	20.3 (17.1–24.0)	18.3 (14.4–23.0)	25.0 (22.0–28.2)*	22.1 (19.4–25.1)	<0.001
Left ventricular hypertrophy	15.3 (10.1–22.5)*	25.8 (19.9–32.7)	27.8 (20.4–36.5)	32.6 (25.6–40.5)	28.9 (22.7–36.0)	<0.001
Microalbuminuria	9.1 (7.7–10.9)*	19.7 (17.5–22.2)	24.1 (19.5–29.4)	26.7 (22.1–32.0)	23.1 (20.3–26.1)	<0.001
Moderate chronic kidney disease	1.4 (0.8–2.5)	3.9 (2.4–6.5)	2.9 (1.2–6.8)	9.3 (5.9–14.4)*	6.1 (3.7–9.9)	<0.001
Severe chronic kidney disease	0.0 (0.0–0.1)	0.1 (0.0–0.4)	0.1 (0.0–0.6)	0.5 (0.3–0.8)	0.3 (0.2–0.5)	<0.001
Ankle-brachial index <0.9	4.1 (2.1–7.7)	5.3 (3.8–7.4)	5.6 (2.6–11.5)	7.2 (3.6–13.8)	6.2 (3.8–9.8)	0.008
At least one asymptomatic HMOD	27.7 (26.5–28.8)*	52.8 (50.3–55.3)	54.5 (49.0–60.1)	64.7 (62.3–66.9)	58.0 (56.4–59.6)	<0.001
Established CV disease						
Stroke	0.2 (0.1–0.5)*	0.3 (0.1–0.8)*	1.4 (0.5–3.5)	3.7 (1.5–8.7)*	1.8 (0.8–4.0)	<0.001
Coronary artery disease	0.3 (0.2–0.6)*	0.4 (0.2–0.8)*	1.3 (0.6–3.1)	2.4 (1.4–3.9)	1.3 (0.8–2.1)	<0.001
Heart failure	1.1 (0.8–1.6)	1.5 (0.6–3.3)	1.6 (0.7–3.5)	2.6 (1.7–4.1)	2.0 (1.1–3.5)	0.024
Atrial fibrillation	0.3 (0.2–0.7)	0.7 (0.4–1.3)	0.8 (0.3–2.0)	1.5 (1.1–2.2)	1.1 (0.8–1.5)	<0.001
At least one established CV disease	1.9 (1.5–2.3)	2.7 (2.1–3.5)	4.7 (3.1–7.0)	9.5 (8.2–11.0)*	5.8 (5.1–6.5)	<0.001

All values were weighted to represent the total Chinese population aged 35 years or older based on the 2010 Chinese census data. Data are represented as percentage (95% CI).

*CV* cardiovascular, *HMOD* hypertension-mediated organ damage.

*P < 0.05, hypertensive patients not on antihypertensive medicines as the reference group.

^#^Compared between participants with normal blood pressure and hypertension.

**Fig. 1:**
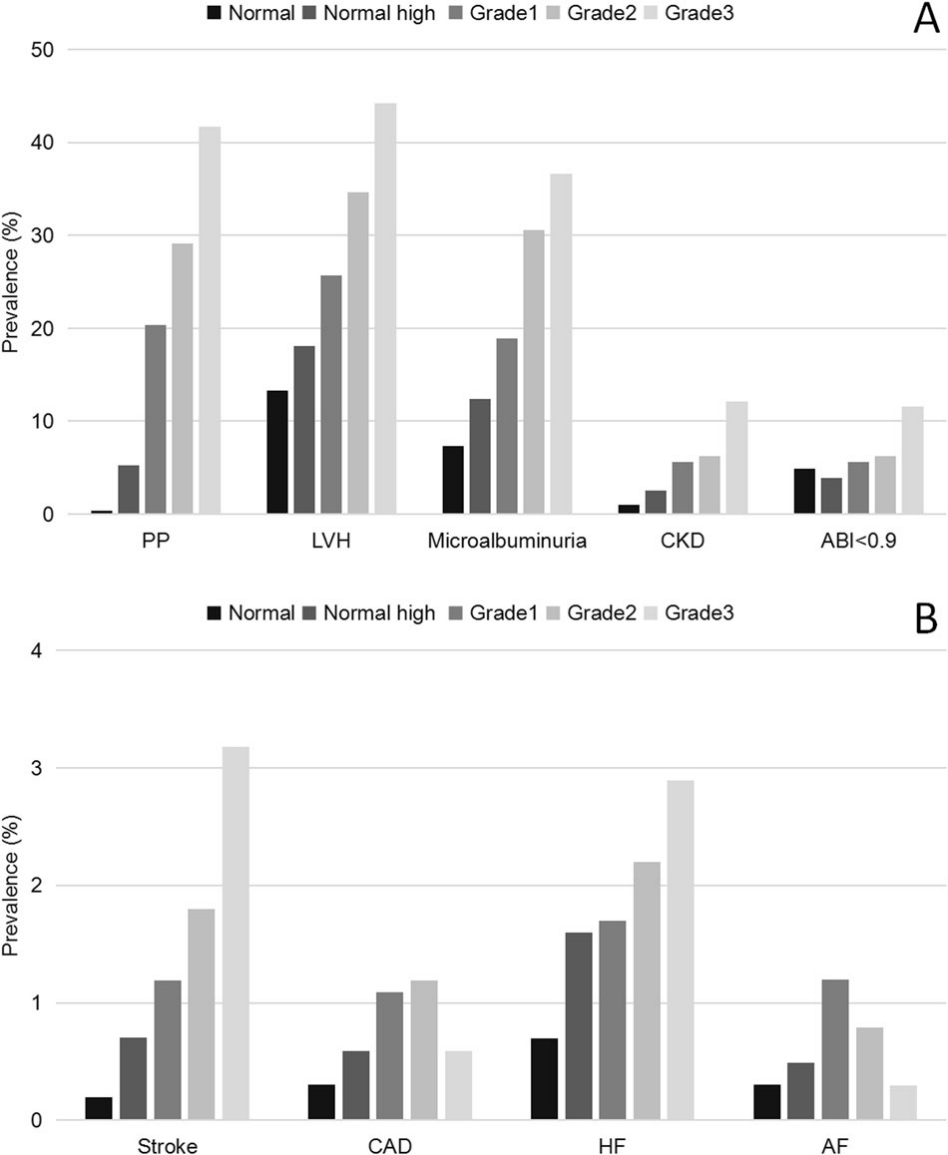
The weighted prevalence of hypertension-mediated organ damage and cardiovascular diseases in hypertensive patients by hypertension grades. **A** The weighted prevalence of hypertension-mediated organ damage in hypertensive patients by hypertension grades. **B** The weighted prevalence of cardiovascular diseases in hypertensive patients by hypertension grades. PP pulse pressure, LVH left ventricular hypertrophy, CKD chronic kidney disease, ABI ankle-brachial index, CAD coronary artery disease, HF heart failure, AF atrial fibrillation. Blood pressure was categorized as normal (SBP < 120 and DBP < 80), normal high (SBP 120–139 and/or DBP 80–89) in normal population, Grade 1 (SBP 140–159 and/or DBP 90–99), Grade 2 (SBP 160–179 and/or DBP 100–109), and Grade 3 (SBP ≥ 180 and/or DBP ≥ 110) in patients with hypertension according to the 2010 Chinese guidelines [15].

**Fig. 2:**
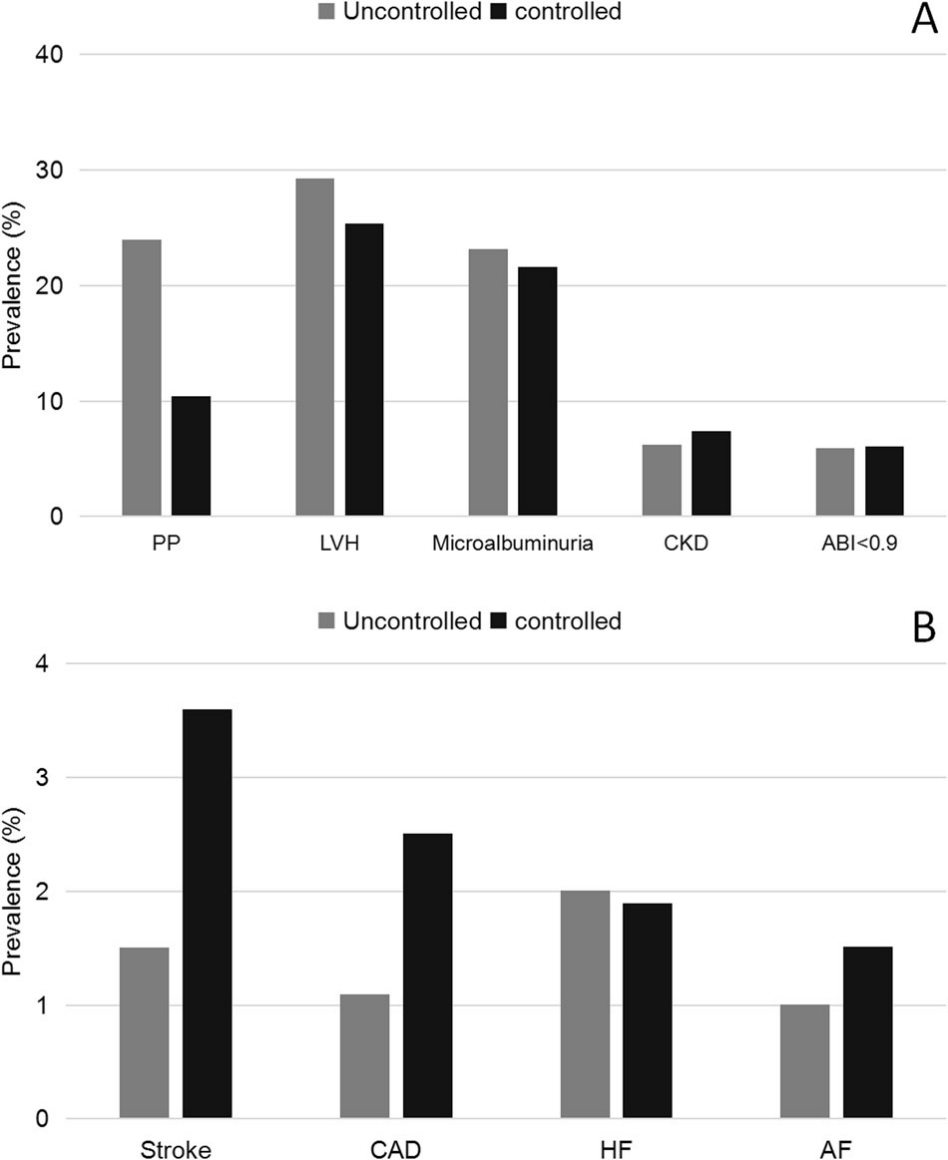
The weighted prevalence of hypertension-mediated organ damage and cardiovascular diseases in hypertensive patients by blood pressure control status. **A** The weighted prevalence of hypertension-mediated organ damage in hypertensive patients by blood pressure control status. **B** The weighted prevalence of cardiovascular diseases in hypertensive patients by blood pressure control status. PP pulse pressure, LVH left ventricular hypertrophy, CKD chronic kidney disease, ABI anklebrachial index, CAD coronary artery disease, HF heart failure, AF atrial fibrillation. Hypertension control was defined as SBP < 140 mmHg and DBP < 90 mmHg in patients.

The prevalence of asymptomatic HMOD, as well as established CVD, was greater for them with higher BP level (*P* < 0.05), except for ABI (Fig. 1). Compared to those whose BP was not under control, the prevalence of wide pulse pressure, LVH, and microalbuminuria were lower in patients whose BP was under control (24.1% vs. 10.8%, 29.5% vs. 25.5%, and 23.3% vs. 21.8%, respectively; all *P* < 0.05). The difference of CKD (6.2% vs. 7.5%; *P* = 0.148) and abnormal ABI (6.1% vs. 6.2%; *P* = 0.929) did not reach the statistically significant between controlled and uncontrolled hypertension groups. For those with established CVD, we found that only the prevalence of HF was lower in patients with controlled hypertension compared to those with uncontrolled hypertension (2.0% vs. 1.9%; *P* = 0.936, Fig. 2).

**Fig. 3:**
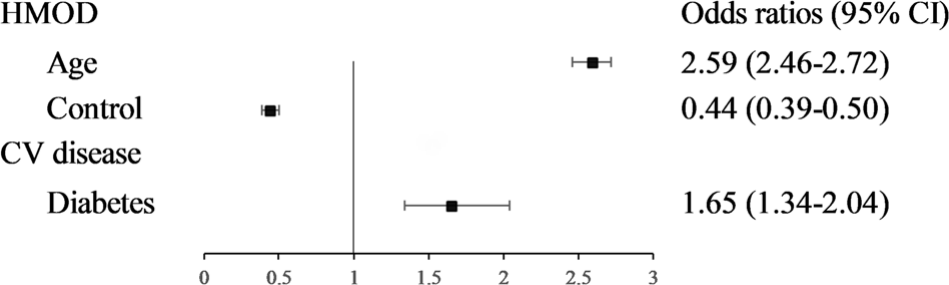
The characteristics highly related to the HMOD and CV diseases. HMOD hypertension-mediated organ damage, CV cardiovascular.

The characteristics significantly associated with HMOD and CVD were different, as shown in Fig. 3. Older adults were more likely to have HMOD.

## Discussion

This study reported the nationally representative estimates of HMOD and established CVD in hypertensive patients. Our study showed that the presence of HMOD in Chinese adults aged 35 or older was common, especially LVH and microalbuminuria. HF was the most common disease complicated with hypertension. Overall, the prevalence of asymptomatic HMOD was lower in patients with controlled hypertension than those with uncontrolled hypertension.

LVH is the most frequent end-organ damage associated with high BP, and it increases the risk of congestive heart failure, sudden cardiac arrest, angina pectoris, myocardial infarction, and cerebrovascular disease [22, 23]. In a meta-analysis of 30 studies on echocardiographic LVH prevalence found that, among 37,700 patients with hypertension, the prevalence of LVH was ~36% of the pooled population according to a conservative criteria [24]. In previous regional studies in China, the prevalence of LVH was between 20.2 and 42.8% among the community-based hypertensive population [25, 26]. Our study showed that the prevalence of LVH was in line with the previous studies (28.9%) and therefore suggests there may be benefitted to widely screening for LVH in hypertensive patients by ECG or, preferably, echocardiography. If necessary, antihypertensive drugs could be used to lower BP and attenuate LVH, for example angiotensin-converting enzyme inhibitors and angiotensin receptor blockers [8].

Microalbuminuria has been recognized as an early sign of renal damage, and increased microalbuminuria indicates endothelial dysfunction or developing atherosclerosis, and predicts end-organ damage, major CV events, and death [27–29]. The prevalence of microalbuminuria in patients with hypertension was less consistent among different study populations [30]. The result was comparable with our previous study [31]. However, the China National Diabetes and Metabolic Disorders Study reported that the prevalence of microalbuminuria in patients with hypertension, but without diabetes, was 37.4% in men and 42.7% in women [32], which was higher than our study. Shandong-Ministry of Health Action on Salt and Hypertension (SMASH) project reported that microalbuminuria was only present in 8.1% of individuals with hypertension [33]. The National Health and Nutrition Examination Survey showed that the average prevalence of microalbuminuria in US adults was 4.5% for normal BP, 6.3% for prehypertension, 12.4% for stage 1 hypertension, 25.3% for stage 2 hypertension, and 11.3% among those with treated, controlled hypertension between 1999 and 2006 [34]. The inconsistent prevalence might be caused by the population structure, definitions of microalbuminuria, and different methods of measurement. Hypertension is a major risk factor for the development and progression of CKD. One meta-analysis showed a significant reduction in all-cause mortality following BP reduction in patients with CKD [35].

The pulsatile component is pulse pressure, which depends on ventricular ejection, arterial stiffness, and timing of wave reflections. Pulse pressure has been shown as an independent risk factor for mortality or CV events in several studies in screening populations or hypertensive patients [36, 37].

Epidemiologic studies have established a strong linear relation between BP and CVD, and randomized trials have documented that BP reductions by antihypertensive drugs against HMOD and established CV disease [38, 39]. Although recent studies have fueled the debate over optimal BP targets in individuals at high-CV risk, they all confirmed the benefits of achieving the target of <140/90 mmHg [40]. Previous studies [41, 42] reported some differences between BP-lowering regimens in their effects on outcomes, and some regimens might be of greater or lesser benefit for patients with established diseases. Taken together, an increasingly important area of hypertension treatment is to identify exiting HMOD or related disease and prescribe an appropriate therapy that could reduce BP and improve patients’ symptoms and prognosis.

We found no significant difference between residents living in urban and rural areas for asymptomatic HMOD and established CV disease in this study. However, the prevalence of wide pulse pressure, CKD, and abnormal ABI were significantly higher in women, while the prevalence of established CVD was higher in men. This finding is in line with majority of previous studies [13, 24, 43].

The current results revealed that the prevalence of HMOD and CVD was higher in the patients on medication. Probably, the medicine use is related to more severe disease, possibly indicating a lack of early screening or late medical intervention in these hypertensive patients. It also may explain the higher prevalence of CVD in patients with controlled hypertension. However, it was not too late that BP control could not play a role in secondary prevention. On the contrary, HMOD was inversely related with BP controlled. It meant early intervention, even without medicine, could delay the progress of damage on the great vessels and the kidney.

This study provided significant contemporary data on the epidemiology of HMOD and established CVD in hypertensive patients in China based on a representative population. One major limitation was that the explanation of the difference between hypertensive patients taking antihypertensive medicines and those not taking antihypertensive medicines should be cautious because we were unable to identify the course of hypertension and severity of hypertension. Another limitation was that the CV events were obtained based on the self-reported history and verified with medical or hospital records, which might lead to underestimation. In addition, the cross-sectional design could not prove a causal relationship. However, as a large survey on HMOD and established CV disease in hypertensive patients, this study still provided significant epidemiological knowledge gaps for middle-income countries.

## Conclusion

In summary, our study showed that the presence of HMOD in Chinese people aged 35 or older was very common. The findings suggest a substantial future burden of both morbidity and mortality from hypertension in China. National strategies should aim at reducing HMOD and established CVD in the population through improving the detection, treatment, and control of hypertension, and through population-based public health interventions to address upstream determinants of health.

## Summary table

### What is known about topic

- Identifying HMOD could help to estimate the burden of high-CV-risk populations in the hypertensive population.
- Earlier screening and intervention in HMOD may lead to more effective therapeutic strategies and reduce future CV events.

### What this study adds

- Determine the prevalence of HMOD and established CVD in a nationally representative population in China.

## Supplementary information

Supplemental
