# Supplementary material for: Hypertension-mediated organ damage and established cardiovascular disease in patients with hypertension: the China Hypertension Survey, 2012–2015
Source: J Hum Hypertens. 2021 Nov 19;36(12):1092–8. doi: 10.1038/s41371-021-00635-z (PMC9734033; doi:10.1038/s41371-021-00635-z)
Supplement: Supplementary file 1 — Supplemental [file 41371_2021_635_MOESM1_ESM.docx]

**Supplementary Tables**

| **STable 1. Participants who were included and those were excluded from the analyses** | | | |
| --- | --- | --- | --- |
| Characteristics | Included | Excluded | *P* value |
| Age (years) | 57.8(57.6-58.0) | 56.9(56.8-57.1) | <0.001 |
| Gender (women) | 55.6(54.8-56.4) | 53.8(53.1-54.4) | 0.001 |
| Body mass index (kg/m^2^) | 24.5(24.5-24.6) | 24.6(24.5-24.6) | 0.228 |
| Rural (%) | 47.2(46.4-48) | 53.8(53.1-54.4) | <0.001 |

| **STable 2. The weighted prevalence of hypertension-mediated organ damage and cardiovascular diseases in hypertensive patients by age group in men** | | | | | | |
| --- | --- | --- | --- | --- | --- | --- |
| **Characteristics** | **35-44 years** | **45-54 years** | **55-64 years** | **65-74 years** | **≥75 years** | ***P* value** |
| **Asymptomatic HMOD** |  |  |  |  |  |  |
| Pulse pressure (in older people) ≥60 mmHg | 0 | 0 | 0 | 71.2(65.3-76.4) | 84(81.8-85.9) | <0.001 |
| Left ventricular hypertrophy | 14.4(7.0-27.4) | 19.3(13.2-27.2) | 24.1(17.2-32.8) | 29.2(21.6-38.2) | 30.2(21.7-40.4) | 0.003 |
| Microalbuminuria | 18.4(12.2-26.7) | 22.4(17.7-27.9) | 17.6(15.1-20.6) | 23.6(20.0-27.6) | 30.2(24.8-36.2) | 0.023 |
| Moderate chronic kidney disease | 1.3(0.4-4.7) | 2.6(0.9-6.7) | 3.3(1.8-5.7) | 10.8(5.9-18.7) | 19.2(11.6-30.1) | <0.001 |
| Severe chronic kidney disease | 0 | 0 | 0.2(0.0-0.8) | 0.2(0.0-0.7) | 0.3(0.1-1.0) | 0.592 |
| Ankle-brachial index <0.9 | 4.0(2.3-6.8) | 3.9(1.9-8.2) | 5.4(2.3-11.9) | 5.7(2.6-11.8) | 8.6(5.0-14.4) | 0.144 |
| At least 1 asymptomatic HMOD | 34(23.6-46.1) | 39.6(32.5-47) | 41.1(33.9-48.7) | 86.2(84.3-88) | 93.2(90.3-95.2) |  |
| **Established CV** **disease** |  |  |  |  |  |  |
| Stroke | 0 | 2.2(0.7-6.7) | 1.8(0.9-3.4) | 4.1(1.8-9.5) | 3.4(1.2-9.3) | 0.125 |
| Coronary artery disease | 0.2(0.0-1.8) | 1.1(0.5-2.4) | 2.6(1.6-4.2) | 2.3(1.2-4.5) | 2.7(1.4-5.3) | 0.007 |
| Heart failure | 1.6(0.4-6.8) | 1.7(0.8-3.2) | 2.2(1.1-4.2) | 2.3(1.4-3.5) | 3.4(1.8-6.4) | 0.395 |
| Atrial fibrillation | 0 | 0.9(0.3-2.4) | 1.9(1.1-3.4) | 1.5(1.0-2.4) | 1.8(1.0-3.5) | 0.021 |
| At least 1 established CV disease | 1.8(0.7-4.7) | 5.6(3.5--8.7) | 7.3(5.4-10.1) | 9.3(7.1-12.0) | 10.0(7.4-13.4) | <0.001 |
| Data are represented as value (95% CI), unless otherwise indicated.  All values were weighted to represent the total population of Chinese aged 18 years or older based on Chinese census 2010. | | | | | | |

| **STable 3. The weighted prevalence of hypertension-mediated organ damage and cardiovascular diseases in hypertensive patients by age group in women** | | | | | | |
| --- | --- | --- | --- | --- | --- | --- |
| **Characteristics** | **35-44 years** | **45-54 years** | **55-64 years** | **65-74 years** | **≥75 years** | ***P* value** |
| **Asymptomatic HMOD** |  |  |  |  |  |  |
| Pulse pressure (in older people) ≥60 mmHg | 0 | 0 | 0 | 83.2(77.1-87.9) | 88.2(84.3-91.3) | <0.001 |
| Left ventricular hypertrophy | 21.2(13.3-32.1) | 32.5(26.6-39.0) | 38.7(30.8-47.2) | 43.3(35.2-51.7) | 47.1(39.5-54.8) | <0.001 |
| Microalbuminuria | 26.8(22.5-31.7) | 20.6(16.5-25.5) | 23.9(19.5-28.9) | 25.6(21.0-30.9) | 35.9(31.6-40.4) | <0.001 |
| Moderate chronic kidney disease | 1.8(1.1-3.1) | 2.5(1.3-4.7) | 4.5(3.0-6.6) | 11.4(6.6-18.9) | 23.9(14.5-36.7) | <0.001 |
| Severe chronic kidney disease | 0.2(0.0-1.7) | 0.1(0.0-0.6) | 0.5(0.2-1.3) | 0.5(0.1-1.9) | 1.4(0.3-5.4) | 0.118 |
| Ankle-brachial index <0.9 | 4.1(2.6-6.4) | 5.7(2.4-12.7) | 7.2(4.4-11.8) | 8.8(5.5-13.7) | 13.5(9.1-19.5) | 0.006 |
| At least 1 asymptomatic HMOD | 46.3(35.7-57.3) | 50.9(46-55.7) | 58.2(53.3-62.9) | 94.7(92.3-96.4) | 96.2(94.1-97.5) | <0.001 |
| **Established CV** **disease** |  |  |  |  |  |  |
| Stroke | 0 | 0 | 2.5(0.9-6.5) | 2.1(1.0-4.6) | 4.2(1.1-15.1) | 0.186 |
| Coronary artery disease | 0.1(0.0-0.7) | 0.8(0.3-2.0) | 1.2(0.5-3.3) | 1.6(1.0-2.6) | 1(0.4-2.9) | 0.118 |
| Heart failure | 0.7(0.1-3.5) | 2.3(1.1-4.7) | 1.6(0.6-3.8) | 1.7(0.7-3.8) | 3.7(2.1-6.5) | 0.090 |
| Atrial fibrillation | 0 | 0.8(0.3-2.6) | 1.0(0.5-2.0) | 1.5(0.9-2.8) | 1.6(0.7-3.5) | 0.130 |
| At least 1 established CV disease | 0.8(0.3-2.4) | 3.9(2.5-6.2) | 6.2(4.6-8.4) | 6.2(4.8-8.2) | 9.8(7.3-13.0) | <0.001 |
| Data are represented as value (95% CI), unless otherwise indicated.  All values were weighted to represent the total population of Chinese aged 18 years or older based on Chinese census 2010. | | | | | | |

| **STable 4. The weighted prevalence of hypertension-mediated organ damage and cardiovascular diseases in hypertensive patients by sex and region (sensitivity analysis)** | | | | | |
| --- | --- | --- | --- | --- | --- |
| **Characteristics** | **Region** | | **Sex** | | **Total** |
|  | **Urban** | **Rural** | **Men** | **Women** |  |
| **Asymptomatic HMOD** |  |  |  |  |  |
| Pulse pressure (in older people) ≥60 mmHg | 20.3(19.2-21.5) | 23.4(22.2-24.7) | 18.1(17.0-19.2) | 26.9(25.6-28.3) | 22.2(21.3-23.1) |
| Left ventricular hypertrophy | 24.3(23.2-25.4) | 34.8(33.7-36.0) | 22.5(21.4-23.6) | 35.7(34.6-36.8) | 29.5(28.7-30.3) |
| Microalbuminuria | 22.0(20.9-23.0) | 22.3(21.3-23.3) | 21.2(20.2-22.2) | 23.0(22.0-23.9) | 22.1(21.4-22.8) |
| Chronic kidney disease | 8.6(7.9-9.2) | 9.0(8.3-9.7) | 8.1(7.5-8.8) | 9.4(8.7-10.1) | 8.8(8.3-9.3) |
| Ankle-brachial index <0.9 | 6.4(5.8-6.9) | 7.0(6.4-7.6) | 6.0(5.5-6.6) | 7.2(6.6-7.8) | 6.7(6.3-7.1) |
| At least 1 asymptomatic HMOD | 50.3(48.3-52.2) | 52.5(50.7-54.4) | 45.7(43.7-47.7) | 58.5(56.7-60.3) | 51.7(50.3-53.0) |
| **Established CV** **disease** |  |  |  |  |  |
| Stroke | 1.3(1.1-1.6) | 1.3(1.0-1.5) | 1.4(1.1-1.7) | 1.2(0.9-1.4) | 1.3(1.1-1.5) |
| Coronary artery disease | 3.2(2.8-3.6) | 0.9(0.7-1.2) | 2.4(2.1-2.8) | 1.8(1.5-2.1) | 2.1(1.9-2.3) |
| Heart failure | 2.9(2.5-3.3) | 2.1(1.7-2.5) | 2.4(2.0-2.8) | 2.6(2.2-3.0) | 2.5(2.2-2.8) |
| Atrial fibrillation | 1.9(1.6-2.2) | 1.4(1.1-1.7) | 1.6(1.3-1.9) | 1.7(1.4-2.0) | 1.7(1.5-1.9) |
| At least 1 established CV disease | 6.4(5.6-7.4) | 3.9(3.3-4.6) | 5.3(4.5-6.1) | 4.5(3.9-5.2) | 4.9(4.4-5.4) |
| Data are represented as value (95% CI) | | | | | |
